# Perturbation Analysis of Heterochromatin-Mediated Gene Silencing and Somatic Inheritance

**DOI:** 10.1371/journal.pgen.1001095

**Published:** 2010-09-09

**Authors:** Jonathan I. Schneiderman, Sara Goldstein, Kami Ahmad

**Affiliations:** Department of BCMP, Harvard Medical School, Boston, Massachusetts, United States of America; Max-Planck-Institute of Immunobiology, Germany

## Abstract

Repetitive sequences in eukaryotic genomes induce chromatin-mediated gene-silencing of juxtaposed genes. Many components that promote or antagonize silencing have been identified, but how heterochromatin causes variegated and heritable changes in gene expression remains mysterious. We have used inducible mis-expression in the Drosophila eye to recover new factors that alter silencing caused by the *bw^D^* allele, an insertion of repetitive satellite DNA that silences a *bw^+^* allele on the homologous chromosome. Inducible modifiers allow perturbation of silencing at different times in development, and distinguish factors that affect establishment or maintenance of silencing. We find that diverse chromatin and RNA processing factors can de-repress silencing. Most factors are effective even in differentiated cells, implying that silent chromatin remains plastic. However, over-expression of the *bantam* microRNA or the *crooked-legs* (*crol*) zinc-finger protein only de-repress silencing when expressed in cycling cells. Over-expression of *crol* accelerates the cell cycle, and this is required for de-repression of silencing. Strikingly, continual over-expression of *crol* converts the speckled variegation pattern of *bw^D^* into sectored variegation, where de-repression is stably inherited through mitotic divisions. Over-expression of *crol* establishes an open chromatin state, but the factor is not needed to maintain this state. Our analysis reveals that active chromatin states can be efficiently inherited through cell divisions, with implications for the stable maintenance of gene expression patterns through development.

## Author Summary

Repetitive DNA and transposons are compacted into heterochromatin in eukaryotic genomes to silence potentially dangerous elements. Heterochromatic silencing is distinct from classical gene repression because affected genes randomly switch on and off during development, with varying degrees of somatic heritability. Here, we focus on the silencing of a reporter gene by a repetitive DNA satellite block on a homologous chromosome. Silencing in this system relies on long-range chromosomal interactions, but these are disrupted during mitosis and must be re-established every cell cycle. We employed an inducible system to identify factors that can alter silencing when over-expressed. The inducible nature of this system allows us to perturb silencing at different development stages, and distinguish factors that affect the establishment or maintenance of silencing. We identified a diverse collection of modifiers, and most can alter silenced chromatin even in differentiating cells. Strikingly, over-expression of one factor – the *crol* zinc-finger protein – establishes a de-repressed state that is somatically heritable. Our analysis of *crol* implicates cell cycle progression in the maintenance of silenced chromatin, and argues that active chromatin can be efficiently propagated through mitotic divisions. Our findings validate inducible modifiers as tools for the dissection of establishment and maintenance of chromatin states.

## Introduction

Eukaryotic DNA is packaged with histones into nucleosomes, which represent the primary unit of chromatin. Nucleosomes render DNA inaccessible to transcription factors, and thus modulate transcriptional activity. Nucleosome stability is governed by chromatin remodeling complexes that move histones with respect to the DNA [1] as well as the physical properties of the sequences the histones wrap [2]. Chemical modifications of histone tails are also important for chromatin transactions, as they affect how nucleosomes interact with each other, recruit auxiliary factors, and define functional chromatin domains [3]. Chromatin can be separated into two types – euchromatin, where most unique genes are found, and heterochromatin, rich in transposable elements and repetitive sequences. While a great deal is known about the different protein composition and signature chemical modifications of these two types of chromatin environments, how they are established and maintained remains mysterious.

Much of our understanding of heterochromatin comes from genetic screens performed with variegating reporter genes in Drosophila. These genetics studies have focused on the repressive effects that heterochromatin exerts on euchromatin when the two are in close proximity, and have identified a number of chromatin factors required for efficient silencing [4], [5]. Molecularly, heterochromatin-mediated silencing is correlated with repressive histone modifications and the association of heterochromatic proteins [6]. Silenced genes exhibit reduced accessibility of restriction enzymes and highly regular nucleosomal arrays, further indicating that repression is achieved through an altered chromatin structure [7]. A silent chromatin state can be established at euchromatin *de novo* by the artificial tethering of heterochromatin factors to a site [8], [9]. However, it remains unknown what the requirements are for the propagation of an altered chromatin state through DNA replication and cell division.

Here we use the GAL4-*UAS* over-expression system [10] to perturb chromatin-mediated silencing. Our analysis reveals a more extensive array of modifiers than previously appreciated. We exploited the modular nature of the GAL4-*UAS* system to address the establishment and maintenance of heterochromatic silencing in cycling and differentiated cells. Our findings indicate that active chromatin states can be established early in development and stably inherited through mitosis, while silenced chromatin is plastic and must be re-enforced every cell cycle.

## Results

**Figure 1:**
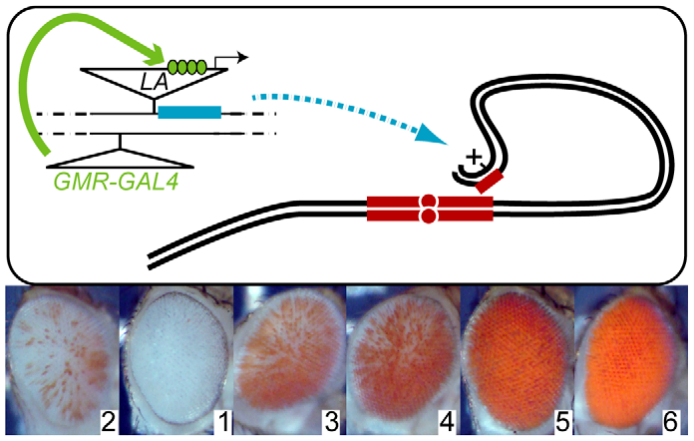
A perturbation screen for *bw^D^*–mediated heterochromatic silencing. (A) New insertions of a *P[LA]* transposon carrying a *y^+^* marker and a GAL4-inducible promoter were recovered in progeny that also carried a eye-specific GAL4 driver (*GMRGAL*) and the *bw^D^* heterochromatic insertion (small red block). GAL4 (green) activates a inducible promoter in the *P[LA]* insertion and transcribes any neighboring gene (blue). Somatic pairing between homologous chromosomes (black lines) and aggregation of heterochromatin (red blocks) normally efficiently silences the paired *bw^+^* eye color gene. We screened for new *P[LA]* insertions that altered *bw^D^* silencing, and identified the position of the *P* element by iPCR. (B) Silencing of *bw^+^* was ranked on a scale from 1 to 6. Normal *bw^D^/bw^+^* silencing was scored as Rank 2, enhancement as Rank 1, and increasing degrees of de-repression as Ranks 3–6. Representative eyes from each rank are shown.

The *brown^Dominant^* (*bw^D^*) allele is an insertion of ∼2 Mb of satellite sequence in the *brown* gene, and confers a heterochromatic chromatin structure to the locus [11]. This insertion causes dominant heterochromatic gene-silencing in *bw^D^/bw^+^* heterozygous adults, so that only ∼5% of eye cells are pigmented [12]. Silencing of the *bw^+^* allele proceeds through a sequence of chromosomal interactions, where the *bw^D^* allele first somatically pairs with *bw^+^*, and then the aggregation of repetitive sequences within the nucleus drags the locus into the heterochromatic chromocenter (Figure 1A; [13]). These interactions are required for silencing of the *bw^+^* gene [14]. Pairing and aggregation are thought to be disrupted every mitosis and frequently reform in each interphase, accounting for the speckled variegation of *bw^+^* silencing in the adult eye [15], [16]. The severity of silencing with *bw^D^* is reliable and consistent between individuals, and we therefore used this system in a screen to recover genes that perturb *bw^D^* silencing when over-expressed.

### Diverse factors modify heterochromatic gene-silencing

We used the modular GAL4-*UAS* mis-expression system [10] to identify endogenous genes that could modify the severity of *bw^D^* silencing when over-expressed in the eye (Figure 1A). We mobilized the mis-expression transposons *P*[*EP*] and *P*[*LA*], both of which contain a GAL4-dependent promoter at one end of the element that transcribes into flanking DNA sequences [10], [17]. New insertions were combined with the eye-specific GAL4 source *GMRGAL* and *bw^D^* to test for effects on heterochromatic silencing, and adults with increased or decreased eye color were retained. We categorized pigmentation of the eye on a scale of 1 through 6, where silencing from the *bw^D^* allele with no mis-expression insertion was assigned a score of 2, and full pigmentation in *bw^+^* adults was a score of 6 (Figure 1B). Insertions with enhanced silencing were assigned a score of 1, and insertions with de-repressed silencing were ranked 3–6 depending on the extent of de-repression.

We recovered 28 *P*[*EP*] modifying insertion lines and 23 *P*[*LA*] insertion lines from ∼1100 fertile individual crosses (Table 1). 45 lines showed de-repression of silencing, and 9 lines showed enhanced silencing. 7 of these lines had effects on eye morphology, but changes in silencing were clear even in these cases where the eye was rough. We used inverse PCR to identify the location of the transposon in each line (Table S1). A single gene could not be identified in most P[*EP*] lines as multiple insertions were present, in part because these lines still carried the donor insertion. The 4 lines with single insertions that could be identified were retained (Table 1; Table S1), and other lines discarded. Inverse PCR successfully identified 22 of the *P*[*LA*] insertions. We also tested candidate over-expression lines from public stock centers that we selected based on molecular pathways known to be involved in heterochromatic gene-silencing, or implicated by hits in our screens (Table 1; Table S2). Each line was verified as requiring GAL4 induction of the flanking genomic sequence for effects on *bw^D^* silencing.

**Table 1:** Over-expression of genes with effects on silencing.

line^a^	CG ID^b^	gene	*bw^D^/bw^+^* silencing^c^				description
			*GMRGAL4*	*eyGAL4*	*A5CGAL4*	*GMR-wIR* d	
EP^Chd12^	*CG3733*	*Chd1*	1	2	viable^g^	++	chromatin remodeler
XPd10097	*CG31212*	*Ino80*	1	1	viable	+++	chromatin remodeler
**LA77A**	*CG1507*	*pur-alpha*	1^e^	2	viable^g^	++	transcription factor
**LA4.5**	*CG11844*	*vig2*	1	3	lethal^h^	++	mRNA-binding
**LA4.4**	*CG10630*	*CG10630*	1	2	lethal	++	mRNA-binding
**LA3.2**	*CG8036*	*CG8036*	1	2	viable	++	transketolase
**LA3.1**	*CG11352*	*Jim*	1	2	lethal	++	zinc-finger protein
**LA5.3**	*CG5486*	*Ubp64E*	1	2	lethal	++	ubiquitin protease
**LAE154**	*CG31868*	*Samuel*	1^e^	2	lethal^h^	++	steroid nuclear receptor
**LA11A**	*CG2368*	*psq*	1^e^	2^f^	lethal	++	transcription factor
no insertion control		–	2	2	viable	++	–
**LA2.1**	*Bte00003*	*Doc*	3e	lethal	lethal	++	retrotransposon
**LA1.4**	*CG8676*	*HR39*	3	2e	lethal	++	steroid nuclear receptor
EP701	*CG5899*	*Etl1*	3	2	viable	n.d.	chromatin remodeler
EY12846	*CG3696*	*kismet*	3	2	viable	++	chromatin remodeler
**LA4.3**	*CG3696*	*kismet*	3	n.d.	n.d.	n.d.	chromatin remodeler
**LAS146**	*CG6930*	*l(3)neo38*	3	2	lethal	++	zinc-finger protein
**LA2.4**	*CG7757*	*CG7757*	3	2	lethal^h^	++	small nuclear riboprotein
**LAS110**	*	*	3	2	viable^g^	++	*
**LA1.3**	*CG5933*	*MTA70*	3	2	viable	++	RNA methyltransferase
**LAJJ2A**	*CG14938*	*crol*	3^e^	2/6^i^	lethal	++	zinc-finger protein
EY09290	*CG9537*	*DLP*	4	2	lethal	++	transcription factor
EY04120	*CG2031*	*Hpr1*	4	2	viable	++	mRNA export factor
**LAS55**	*CR33559*	*bantam*	4^e^	lethal	lethal^h^	++	microRNA
**LA2.5**	*CR33559*	*bantam*	4^e^	n.d.	n.d.	n.d.	microRNA
**LA1.6**	*CG3162*	*CG3162*	4	2	lethal	++	small nuclear riboprotein
EY06795	*CG10279*	*Rm62*	4	2	viable	+	RNA helicase
EY03252	*CG32438*	*SMC5*	4	2	viable	n.d.	condensin
LA00872	*CG9383*	*Asf1*	4	lethal	lethal	n.d.	histone chaperone
XPd04051	*CG8989*	*His3.3B*	4	2	viable	n.d.	histone variant
EY23248	*CG17921*	*HmgZ*	4	3	viable	+	high mobility group protein
EY03609	*CG17950*	*HmgD*	5	2	lethal^h^	+	high mobility group protein
**LA3.4**	*CG5794*	*CG5794*	5	2	viable	++	ubiquitin protease
EP635	*CG4548*	*xnp*	5	4	lethal	++	chromatin remodeler
EY08629	*CG1966*	*Acf1*	6	3	viable	+	chromatin assembly factor
EY10737	*CG33182*	*CG33182*	6	2	viable	n.d.	histone demethylase
**EP14C**	*CG13895*	*CG13895*	6	2	viable	++	CENPB motifs
**LA4.1**	*CG3941*	*pita*	6	2	viable	++	zinc-finger protein
**EPDJ1**	*CG12819*	*sle*	6	2	viable	++	nucleolar protein
**EP27**	*CG13109*	*tai*	6	2^e^	lethal	+++	transcription factor
**EP13**	*CG5935*	*Dek*	6	2	viable^g^	++	chromatin & splicing factor

a – Bold, lines recovered in screens; non-bold, candidate lines.

b – * an genomic insertion site was not identified by iPCR.

c – severity of silencing was scored in ranks from severe silencing (1) to no silencing (6).

d – eye color after w+ knockdown ('++' amount in controls, '+++' increased, '+' reduced).

e – rough eyes.

f – small eyes.

g – lethal in males.

h – pupal lethal.

i – sectors of complete de-repression in a speckled background.

Altogether, we identified 36 genes that act as over-expression modifiers of *bw^D^*. These include 7 genes that are known to be required for heterochromatic gene-silencing from previous studies with null alleles (*psq* [18], *Ubp64E* [19], *ASF1* [20], *Acf1* [21], *xnp* [22], [23], *Rm62* [24], and *vig2* [25]). Some of these factors have also been implicated in Polycomb-dependent silencing, suggesting that this screen may identify factors that can affect multiple levels of chromatin structure and gene expression. As these factors affect silencing when over-expressed, caution is necessary in inferring their normal functions. Indeed, we noted that in many cases over-expression of a factor had similar effects on silencing as null alleles for that factor, suggesting that their effect is not simply due to increased dosage of the factor. We group these factors according to their annotated biological function below.

#### Chromatin factors

A diverse collection of chromatin factors affects *bw^D^* silencing. We recovered insertions that over-express structural components of chromatin (*SMC5*, *HmgZ*, *HmgD*, and *His3.3B*). HmgZ, HmgD, and H3.3 are enriched in active chromatin [26], [27], and might promote the activation of genes when over-expressed. SMC5 has been implicated in both compaction of chromatin and in long-range enhancer-promoter interactions during gene activation. Structural components of chromatin are believed to be important for connections between nucleosomes [28]. A second set of chromatin factors implicates nucleosome assembly and remodeling (the remodelers Chd1, Ino80, Etl1, Kismet, and XNP; the histone chaperones ASF1 and Dek; the chromatin assembly factor ACF1). It is striking that individual remodelers have distinctive effects on silencing, presumably by altering nucleosome dynamics at specific sites in the genome [22]. Finally, we identified one gene with histone modifying activity – the histone JmjC demethylase *Kdm4B*. This demethylase removes methylation from both histone H3-K9 and H3-K36 residues [29], [30]. The insertion *Kdm4B^EY10737^* partially de-represses silencing on its own, but *bw^D^* is further de-repressed with *GMRGAL* induction.

#### Transcription factors

Previous studies have identified mutations in genes encoding transcription factors as modifiers of heterochromatic silencing [31]. These mutations are thought to affect the competition between activation and repression at genes juxtaposed to heterochromatin, thereby enhancing silencing. We identified 10 genes annotated as transcription factors that alter *bw^D^* silencing when over-expressed. Over-expression of *HR39*, *l(3)neo38*, *crol*, *DLP*, *CG13895*, *pita*, *Dek*, and *tai* de-repress silencing, consistent with the idea that excess production of these factors may overcome repressive effects of heterochromatin. In contrast, over-expression of the *psq*, *pur-alpha* *Jim*, and *Samuel* transcription factors enhance silencing. We noted that a number of the recovered factors (*Samuel*, *HR39*, *crol*, *tai*, and *Dek*) are linked to ecdysone hormone-triggered developmental responses. The levels of these proteins change during development, and this suggests that ecdysone responses stimulate global change in heterochromatin. A developmentally-regulated aspect of heterochromatic silencing has been previously suggested from patterns of silencing of a *HS-lacZ* gene [32], [33].

#### RNA processing factors

This group of modifiers includes RNA binding and export factors (*vig2*, *Hpr1*, *Rm62*, *sle, CG10630*), an RNA modification enzyme (*MTA70*), and splicing components (*CG7757*, *CG3162*, *Dek*). The *vig2* and *Rm62* genes have been previously identified as involved in heterochromatic silencing [25], [24]. Our recovery of splicing factors and RNA modifying enzymes implicates additional aspects of RNA metabolism in silencing.

#### Miscellaneous factors

Some factors we identified have domains that only partially identify their functions. We identified the transketolase *CG8036*, and 2 ubiquitin-dependent proteases (*Ubp64E*, *CG5794*). *Ubp64E* has been previously identified as a modifier of silencing, and may modulate the stability of chromatin proteins after ubiquitinylation [19]. Other factors may also act by modifying heterochromatin proteins.

Two remaining factors were surprising because the recovered insertion sites did not map near annotated protein-coding genes. The insertion *P*[*LA*]S55 lies at position 638208 of chromosome *3L*, and *P*[*LA*]2.5 lies nearby at position 639482. Both insertions are upstream of the *bantam* microRNA precursor gene (4 Kb and 2.5 Kb, respectively) and oriented so that GAL4 induction may over-produce this transcript. These insertions appear to generate functional *bantam* microRNAs, because induction by *GMRGAL* produces enlarged eyes in adults, consistent with the role of *bantam* in promoting cell division and growth [34]. A third insertion – *P*[*LA*]2.1 – lies in the 5′ UTR of a *Doc* retrotransposon and maps to the second chromosome. This insertion is oriented to over-produce the *Doc* transcript. However, induction of *P*[*LA*]2.1 probably produces a transcript from an unidentified gene downstream of the *Doc* insertion, because other mis-expression insertions in selected *Doc* elements do not recapitulate the phenotype of *P*[*LA*]2.1 (data not shown).

### Specificity of over-expression for heterochromatic rearrangements

We tested our insertions with a series of additional assays. We first determined if over-expression modifiers have general effects on heterochromatic silencing, or are limited to *bw^D^*-mediated silencing. As an independent test of silencing, we used the inversion *In(1)w^m4^* (*w^m4^*) where the *w^+^* gene is juxtaposed to pericentric heterochromatin. Many mis-expression constructs carry a *w^+^* marker and cannot be assayed with *w^m4^*. However, the *P*[*LA*] element we used is marked with *y^+^*; thus we could induce our lines using *GMRGAL* in combination with *w^m4^* and then assess effects on *white* silencing. We found that all 8 enhancers of *bw^D^* silencing are also enhancers of *w^m4^* silencing (Table 2). This implies that these factors do indeed have general effects on heterochromatin. The effects of *bw^D^* de-repressors are more variable. Only 1 line de-represses both *bw^D^* and *w^m4^*, and 9 lines have no effect on *w^m4^*. Surprisingly, 2 lines de-repress *bw^D^* but enhance *w^m4^*. Previous studies have also found that the *bw^D^* and *w^m4^* rearrangements are not equivalently affected by all modifiers of heterochromatic silencing [35]. These differences suggest that each chromosome rearrangement has a unique combination of gene regulatory elements and heterochromatic sequences that determine the extent of silencing. The testing of *bw^D^* modifiers for effects on silencing of *w^m4^* is informative, as it reveals that enhancers are general, yet de-repressors are not. The silencing of *bw^+^* by *bw^D^* provides a sensitive assay for multiple levels of chromosomal organization. Over-expression lines that perturb *bw^D^* silencing yet have no effect on *w^m4^* may affect *bw^+^* regulation, chromosome pairing, or heterochromatic aggregation, all of which are required for silencing in *trans*.

**Table 2:** Specificity of modifiers for heterochromatic rearrangements.

gene	silencing^a^	
	*bw^D^/bw^+^*	*w^m4^*
*pur-alpha*	1	E
*vig2*	1	E
*CG10630*	1	E
*CG8036*	1	E
*Jim*	1	E
*Ubp64E*	1	E
*Samuel*	1	E
*psq*	1	E
		
*LA2.1*	3	E
*HR39*	3	N
*l(3)neo38*	3	S
*CG7757*	3	E
*LAS110*	3	N
*MTA70*	3	N
*crol*	4	N
*bantam*	4	N
*CG3162*	4	N
*CG5794*	5	N
*pita*	6	N
*LAS154*	6	N

a – Severity of *bw^D^* silencing in indicated by ranks (1, severe to 6, de-repressed); severity of *w^m4^* silencing as S (suppressed), E (enhanced) or N (no effect).

### Over-expression modifiers do not affect RNAi

Functional RNAi systems are required for heterochromatic silencing in eukaryotes. Nuclear complexes containing small RNAs are thought to target histone modifications to homologous repetitive sequences, and to promote the retention and subsequent degradation of nascent transcripts from those repeats [36]. In Drosophila, the RNA endonuclease Dcr2 is required for both production of post-transcriptional silencing small RNAs and for small RNAs derived from transposable elements in the genome [37]. Genetic evidence in Drosophila indicates a link between RNAi and heterochromatic silencing as well [38]–[40], although the mechanism of how RNAi is converted into chromatin structure has not been detailed. We recovered 10 lines that are implicated in RNA metabolism in our screen (Table 1), and we tested whether over-expression lines might affect *bw^D^* silencing by inhibiting RNAi. We used a hairpin construct that eliminates *w^+^* message (*GMR-wIR*) to assay the effectiveness of RNAi [41]. *GMR-wIR* eliminates almost all pigmentation in the eye, and this effect requires Dcr2 activity. As the hairpin construct and the over-expressed modifiers are both induced from *GMR* promoters, the double-strand RNA for knockdown and the modifier are expressed at the same time starting late in the development of the eye.

There were no significant effects of over-expression lines on the extent of *w^+^* knockdown by *GMR-wIR*, although 3 lines showed a slight increase in eye pigmentation, and 4 lines had a slight decrease (Table 1, Figure S1). In these 7 lines the mis-expression *P* elements carry a *w^+^* marker, and the marginal effects on knockdown may be due to different levels of *w^+^* expression. Regardless, the severe effect of these insertions on heterochromatic silencing with little effect on knockdown implies that these factors do not affect silencing by altering RNAi.

### Most factors require high-level expression to affect silencing

The *GMRGAL* driver we used in our screen produces high levels of GAL4 late in eye development, immediately before the last S phase and cell division of pigment cells in the 3^rd^ instar imaginal disc [42]. Previous experiments have indicated that heterochromatic silencing varies during the development of the eye [43]. We used the modular nature of the GAL4-*UAS* system to test if continual production of factors in the eye would also alter silencing. The *eyGAL* driver produces moderate levels of GAL4 in the eye primordium starting in embryogenesis, and shuts off just before the last cell division in the developing eye [44]. We anticipated that factors may only be effective when expressed with *GMRGAL* if they are required at high levels to modify silencing. Indeed, we found that the majority (29/34) of lines have no effect on silencing when induced by *eyGAL* (Table 1).

Only five factors affected *bw^D^* silencing when induced by *eyGAL4* (Table 1). Induction of the chromatin remodelers *xnp* and *Ino80* have quantitatively similar effects on silencing whether they are induced by *GMRGAL* or by *eyGAL*. This implies that moderate expression of these factors is sufficient for their effect. *GMRGAL* induction of *ACF1* has a dramatic de-repression of silencing, but de-repression is more moderate with *eyGAL*, suggesting that amounts of ACF1 are limiting for de-repression. Late induction of *vig2* enhances silencing, but early induction de-represses silencing. Vig2 is normally produced early in development and may promote the formation of heterochromatin, while the related Vig protein may take over its functions in later development [25]. Perhaps early over-expression of Vig2 interferes with function in early development, while later expression interferes with Vig function. Finally, *crol* is an exceptional case, because over-expression with *eyGAL* gives more dramatic de-repression than induction by *GMRGAL*. This line is examined in more detail below.

The effects of early over-expression on silencing could not be determined for 5 lines that are lethal or severely distort the eye in combination with *eyGAL* (Table 1). These appear to be cases where continuous expression is toxic to cells. It is notable that toxic effects are infrequent in this collection of modifiers. Indeed, when we ubiquitously induced modifier lines with the *A5CGAL* driver, 19 had no effect on viability (Table 1). This includes seven lines with strong de-repressive effects on silencing when induced by *GMRGAL*. This is consistent with the observation that some modifiers of heterochromatin silencing are largely dispensable for viability in Drosophila [45]. Lines that are lethal when constitutively expressed are likely to have more general effects on chromatin regulation.

### *O*ver-expression of *crol* leads to heritable de-repression

**Figure 2:**
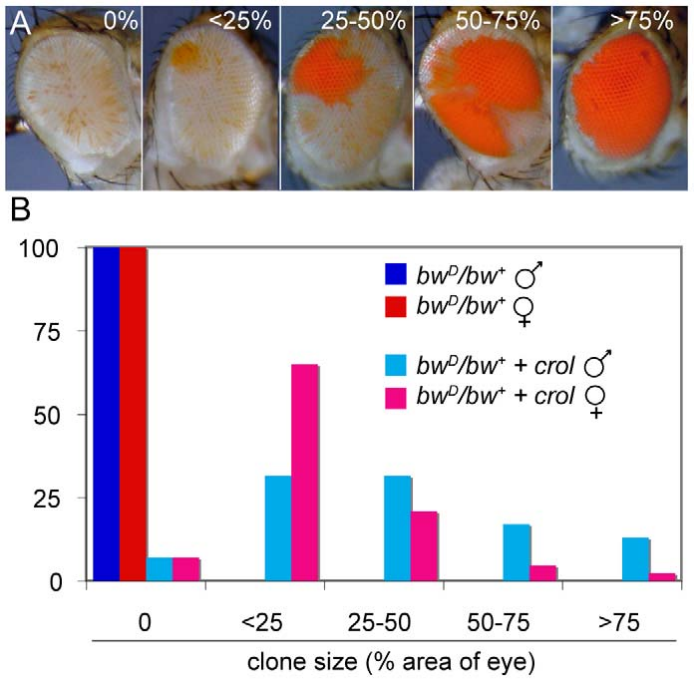
Early over-expression of *crol* leads to sectored de-repression of *bw^+^*. (A) Early induction of the transcription factor *crol* with the *eyGAL4* driver leads to sectors of complete de-repression in a *bw^D^/bw^+^* background. Eyes were assigned to 5 ranks based on the percentage of the area of the eye included in de-repressed sectors. (B) The percentage of eyes with de-repressed sectors in males and females with *bw^D^* and over-expressing *crol* is shown (p<10^−57^ between control and *crol*-expressing males; p<10^−9^ between *crol*-expressing males and females). At least 100 animals were scored for each genotype.

The *crol* transcription factor is one of the few factors tested that de-represses silencing when continually expressed in the eye (Table 1). Strikingly, continual expression of *crol* changes the pattern of silencing in *bw^D^/bw^+^* animals (Figure 2A). The *bw^D^* allele normally causes speckled variegation of *bw^+^* that is thought to result from the disruption and re-establishment of inter-chromosome interactions every cell cycle as the eye grows [15], [16]. However, *bw^D^/bw^+^* animals with continual expression of *crol* frequently have de-repressed sectors in the eye. Most animals with *crol* expression show one or more sectors, implying that de-repression is frequent in this genotype (Figure 2B). Sectors appear in a speckled background, implying that *bw^D^* silencing remains severe for some cells.

**Figure 3:**
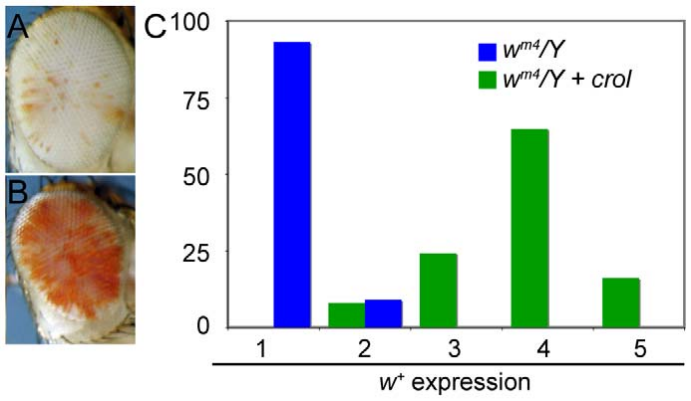
Early over-expression of *crol* is a general de-repressor of heterochromatic silencing. (A) The *w^+^* gene in *w^m4^/Y; eyGAL/+* males show severe silencing. (B) In *w^m4^/Y; eyGAL/crol^JJ2A^* males the *w^+^* gene is de-repressed. (C) Eyes from male flies were assigned ranks based on the pigmented area (1, no pigment to 5, mostly pigmented), and the percentage of eyes with w+ expression with and without *crol* over-expression is shown (p<10^−14^). At least 40 animals were scored for each genotype.

We verified that de-repressed sectors were due to *crol* expression using an independent over-expression insertion line (*d03228*, Exelexis Stock Center). Furthermore, increased *crol* expression with two *eyGAL4* drivers also increases the frequency of de-repressed sectors. Continual expression of *crol* also de-represses *w^m4^* (Figure 3), indicating that this factor can generally modify heterochromatic silencing. Late induction of *crol* moderately de-represses silencing with *bw^D^* and has little or no effect with *w^m4^*, implying that the timing of *crol* expression during development is important for de-repression.

**Figure 4:**
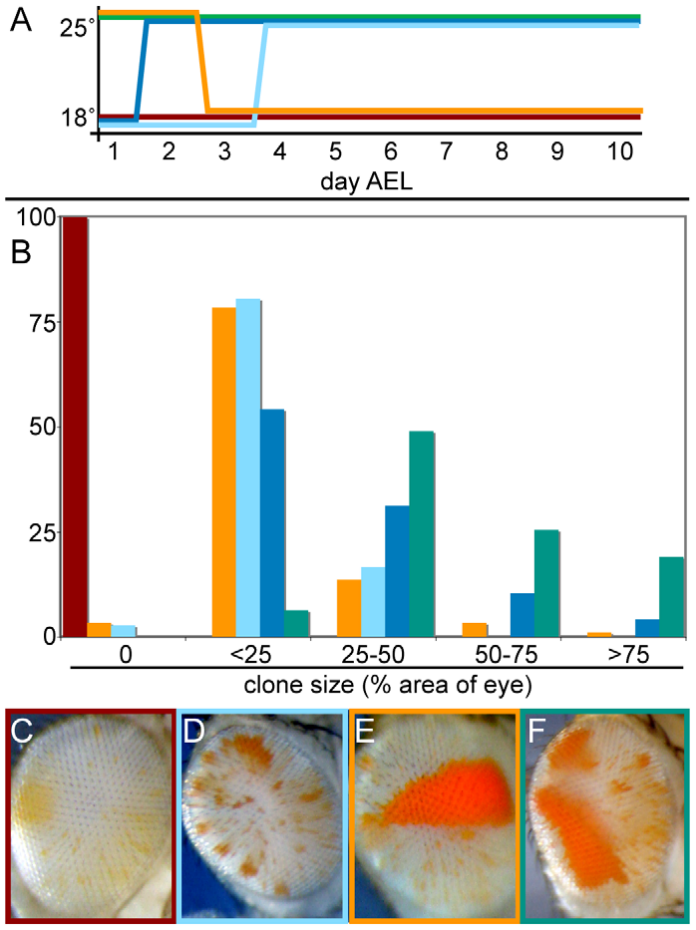
Transient *crol* expression establishes heritable de-repression. (A) Scheme for temperature shifts from 18°C to 25°C and vice versa. Each colored line represents a temperature regimen after egg collections from *eyGAL bw^D^; st* x *crol^JJ2A^; st* crosses, and the proportion of clonal de-repression was counted in male progeny. Flies were also raised continuously at 25° (green line) and 18° (red line) as controls. (B) The percentage of eyes with de-repressed sectors for each temperature regimen in (A) is shown. 40–100 animals were scored for each regime. (C–F) Distinctive eyes of animals raised in the temperature regimes indicated by color lines in (A). (C) Development at 18° inhibits de-repression by *crol* over-expression. (D) Some animals raised at 25° for 2–3 days and then shifted to 18° show single early sectors. (E) Animals raised at 18° for 2–3 days and then shifted to 25° show numerous small sectors. (F) Animals raised at 25° show multiple large de-repressed sectors.

The sectored pattern of variegation suggested that continual *crol* expression causes somatically heritable de-repression. To test this idea, we reduced the strength of *crol* over-expression by raising animals at 18°C, where GAL4 is less effective as an activator [46]. Indeed, raising animals at 18° completely blocks the appearance of de-repressed sectors (Figure 4A–4C). This allows us to use temperature shift experiments to determine the developmental timing when crol causes de-repression. We found that animals raised at 18° for early development and then shifted to 25° showed reduced de-repression (Figure 4A and 4B). Strikingly, some animals raised in this regimen showed numerous small sectors (Figure 4D), consistent with the idea that de-repression does not occur early in this regime but often occurs in later development. This idea is supported by our observation that animals shifted to 25° after 1–2 days at 18° show more de-repression than animals shifted to 25° after 3–4 days (Figure 4B). We conclude that *crol*-stimulated de-repression can occur sporadically throughout development.

To determine if *crol* over-expression is required for the establishment of de-repression, for its stable inheritance, or for both, we transiently expressed *crol* early in development. We raised animals at 25° for embryonic and early larval stages, and then shifted them to 18°. De-repression persisted in this temperature regimen and often appeared as a single sector in the eye (Figure 4E), demonstrating that de-repression can be maintained in the absence of *crol* expression. Thus, the strong effect of continual *crol* expression appears to result from multiple de-repression events throughout development (Figure 4F). We conclude that *crol* is required for de-repression, but de-repression can be maintained through cell divisions without over-expression of the factor.

### Accelerated cell cycles de-repress silencing

**Figure 5:**
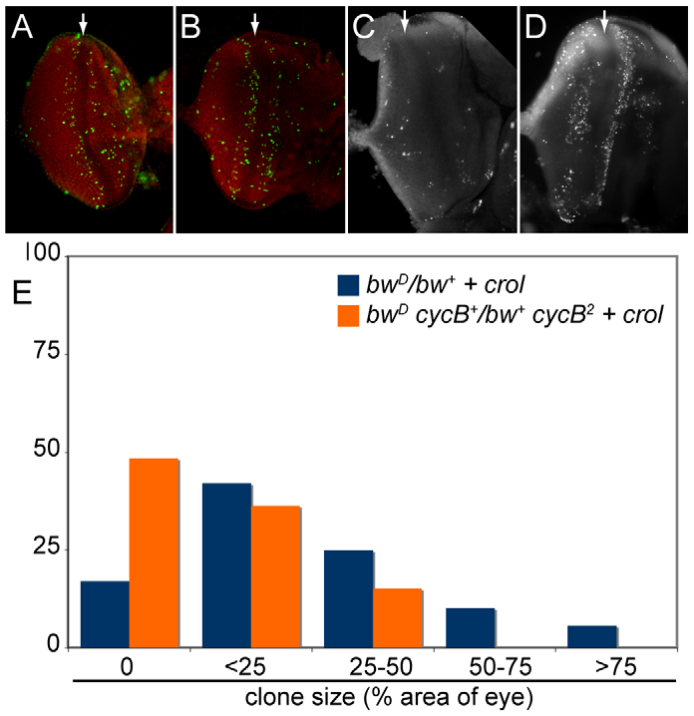
Accelerated cell cycles accompany *crol* -mediated de-repression. (A) Mitotic cells (H3-phospho-S10 staining, green) are detected on both sides of the morphogenetic furrow (MF, arrow) in wildtype eye imaginal discs. (B) Over-expression of *crol* by the *eyGAL* driver increases the number of mitotic cells in eye imaginal discs. 10 discs for each genotype were examined (p<0.04). (C) Wildtype discs show a small number of apoptotic cells (acridine orange staining). (D) Over-expression of *crol* stimulates cell death in the mitotically active regions on either side of the MF. 10 discs for each genotype were examined (p<0.004). (E) The percentage of eyes with de-repressed sectors in males over-expressing *crol* with or without a heterozygous *cycB^2^* mutation is shown. The *cycB^2^* allele significantly reduces sectored de-repression (p<10^−5^). 60–90 animals were scored for each genotype.

How does *crol* de-repress silencing? We noted that *GMRGAL*-induced *crol* expression resulted in a slight roughening of the eye, suggesting that there may be proliferation defects. Indeed, over-expression of *crol* promotes cell division in developing wing discs [47]. We confirmed that *crol* over-expression also promotes cell cycle progression in eye discs. In late third instar larvae, the eye disc contains both mitotically active cells and differentiating cells, and the last two waves of cell divisions in the eye occur on either side of the morphogenetic furrow (MF; [48]). Over-expression of *crol* causes a substantial increase in the number of mitotic cells on both sides of the MF (Figure 5A and 5B). This is accompanied by increased cell death in these zones (Figure 5C and 5D). Previous studies have shown that increased cell proliferation induces compensatory cell death in developing imaginal discs [49]. Thus, *crol* induces both accelerated cell cycles and stable de-repression of silencing in the developing eye.

Acceleration of the cell cycle by *crol* over-expression is suppressed by mutations the mitotic regulator *cyclin B* (*cycB*; [47]). We used this to test if cell cycle acceleration by crol causes de-repression. We found that *cycB^2^* dominantly reduces de-repression by *crol* over-expression (Figure 5E). We conclude that de-repressed clones result from an acceleration of the cell cycle. Notably, *cycB* mutations have no dominant effect on *bw^D^* silencing, demonstrating that silencing and clonal de-repression are genetically distinct processes.

### De-repression by *crol* and *bantam* is limited to cycling cells

**Figure 6:**
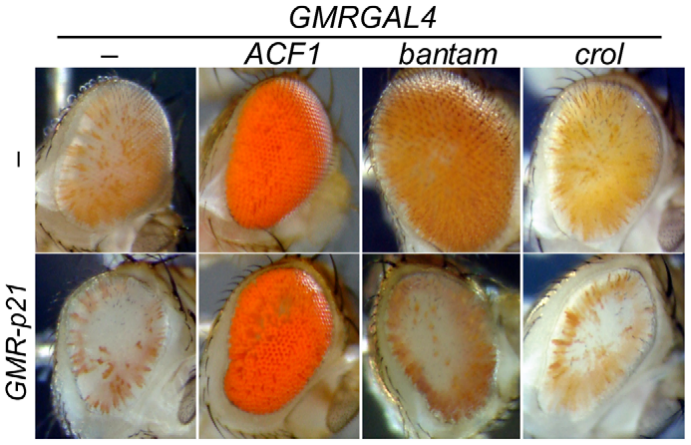
Cell cycle requirements for inducible de-repression. The *GMR-p21* construct blocks Cyclin E activity and eliminates the last cell division in the eye. Flies with *GMR-p21* have slightly reduced and roughened eyes, but still show efficient silencing by *bw^D^*. *GMRGAL4*-induced expression of *ACF1* strongly de-represses silencing, and this is not affected by *GMR-p21*. In contrast, de-repression of silencing by *crol* or *bantam* over-expression is abrogated by a contemporaneous expression of *p21*. At least 5 animals were scored for each genotype.

If accelerated cell cycles induced by *crol* over-expression cause de-repression, then *crol* over-expression in post-mitotic cells should have no effect on silencing. The *GMRGAL* driver induces transgenes immediately before the last cell division in the eye, and induction of *crol* with this driver moderately de-represses *bw^D^* silencing. We used the cyclin inhibitor p21 to eliminate the last division in the eye disc [50]; in this background *GMRGAL* induces *crol* after the last cell division. We found that eliminating the last cell cycle blocks the de-repressive effect of *crol*, confirming that *crol* over-expression is only effective in cycling cells (Figure 6).

A second factor we identified also implicated cell cycle progression in de-repression. The *bantam* microRNA promotes cell growth, and indeed, late over-expression of this factor with *GMRGAL* leads to both de-repression of silencing and expansion of the eye (Figure 6). To determine whether the de-repressive effects of *bantam* are also limited to cycling cells, we tested if de-repression could occur when p21 was also expressed. We found that eliminating the last cell cycle greatly reduces de-repression caused by *bantam* over-expression (Figure 6).

Finally, we tested if other over-expression modifiers are also only effective in mitotically active cells. We focused on the 9 lines that show dramatic de-repression (Rank 6 in Table 1). All 9 of these factors show dramatic de-repression when expressed either before or after the last division in the eye (Figure 6). We conclude that the silenced chromatin state is plastic, and over-expression can overcome silencing even in differentiated cells. The *bantam* and *crol* factors are exceptional, in that they are only effective in cycling cells.

### Establishment is distinct from silencing

**Figure 7:**
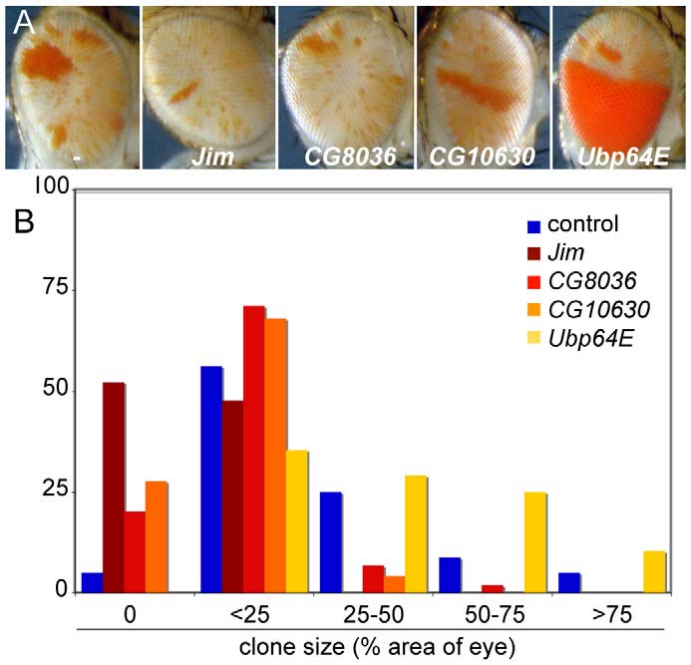
Inducible enhancers alter *crol*-mediated establishment of de-repression. (A) Representative eyes showing heterochromatic silencing with the early expression of *crol* contemporaneous with the indicated inducible enhancer. The size and frequency of de-repressed clones is altered by each enhancer, but the background speckled variegation is unchanged. (B) Histograms of the area of the eye included in de-repressed sectors (p<10^−3^ for all 4 enhancers, at least 45 animals were scored for each genotype).

Over-expression of *crol* generates bw^+^ clones, implying that some cells establish de-repressed early in development and their daughter cells maintain de-repression. We used inducible modifiers we recovered to test if enhancers could inhibit establishment or the maintenance of crol-mediated de-repression. *Jim, CG8036, CG10630*, and *Ubp64E* are enhancers of silencing when induced late in development, but have no effect when expressed early (Table 1). To test if these factors affect clonal de-repression, we induced each of these factors and *crol* early in development. While *crol* induction results in extensive clonal de-repression, contemporaneous expression of *Jim, CG8036*, or *CG10630* strongly reduced clones (Figure 7A and 7B), suggesting that establishment of de-repression is more sensitive than *bw^+^* expression later in development. In contrast, contemporaneous expression of crol and Ubp64E dramatically increases clonal de-repression in the eye (Figure 7B), implying that more cells sporadically switch to a de-repressed state. Thus, while the establishment of de-repression is sensitive to some modifiers of heterochromatic silencing, these appear to be genetically distinct.

## Discussion

We used an efficient over-expression screen to recover dominant modifiers of heterochromatin-mediated gene silencing. The inducible GAL4-*UAS* system allows us to limit over-expression from insertion elements to the eye, thereby avoiding potential toxic effects, as well as testing factors that may not be normally expressed in this tissue. Our screen identified a diverse set of 36 factors that are effective for enhancing or de-repressing silencing, including 7 factors have been previously implicated in heterochromatic function. Some of these factors are likely to directly affect heterochromatin structure, while other factors may have more indirect effects. However, the inducible feature of these modifiers allows us to manipulate heterochromatic silencing by controlling the timing and level of modifier expression. Our results show that both the active and the silenced chromatin states are plastic and epigenetic, as they can be reversed even in post-mitotic differentiating cells. Furthermore, inducible control of modifiers allows us to distinguish between establishment and maintenance of silencing during development.

Patterns of variegation are characteristic of individual chromosomal rearrangements that cause silencing. Silencing due to the *bw^D^* insertion shows a fine-grained speckled pattern of variegation, and the lack of clonal variegation implies that this rearrangement cannot propagate silenced chromatin state. Long-range interactions between heterochromatic regions within the nucleus are required to silence *bw^+^*, and heterochromatic aggregation is thought to be disrupted every cell division. Thus, every daughter cell must re-establish silencing anew after cell division, and even though silencing by *bw^D^* is highly efficient, disruption of heterochromatic interactions every mitosis limits the somatic heritability of silencing. Sporadic speckling where ∼5% of pigment cells have *bw^+^* expression is therefore a result of rare and independent de-repression that occur late in eye development.

In spite of this instability, over-expression of the *crol* transcription factor efficiently de-represses silenced genes, and daughter cells then maintain de-repression. Clonal gene activity in an otherwise silenced population of cells requires that the de-repressed chromatin state be heritable through multiple rounds of mitotic divisions. The speckled variegation of *bw^D^/bw^+^* makes it surprising that expression of a single factor can confer a somatically heritable state to this genotype. It is likely that the crol factor perturbs a normal process carried out by cells, resulting in an inability to silence.

The *de novo* generation of de-repressed and stable clones in unstable silencing system has implications for the mechanism of heterochromatic silencing. Our experiments show that expression of *crol* can establish a heritable de-repressed state as early as embryogenesis, but the *bw^+^* gene is not expressed until eye differentiation ∼5 days later [51]. Thus, the heritable state must be established independently of the expression state of *bw^+^* gene. This distinction has been previously demonstrated for a number of gene activation models, where the establishment of an accessible chromatin state precedes gene activation. For example, the beta-globin locus becomes “open” before transcription initiates in erythrocyte cells [52], [53]. Similarly, monoallelically-expressed loci have “open” and “closed” chromatin features many cell divisions before transcription of one allele begins, and the activated allele is always the “open” locus [54]. Open and closed states of chromatin may correspond to histone modifications that recruit chromatin factors. Our experiments suggest that over-expression of *crol* induces such an open chromatin state at *bw^+^*, thereby permitting its expression later in development.

Crol is a zinc-finger protein that binds chromatin [47], and may directly contribute to inducing an open chromatin state. However, its effect on heterochromatic silencing requires acceleration of the cell cycle, and silencing is restored when cell cycle progression is either delayed with *cyclin B* mutations or blocked with cyclin E inhibitors. Our identification that the proliferation-inducing *bantam* microRNA also de-represses silencing confirms that cell cycle progression affects *bw^D^* silencing. There is extensive evidence that cell cycle progression is generally important for heterochromatic silencing. New nucleosome assembly during the duplication of chromatin in S phase dilutes histone modifications localized in the genome. The dilution of heterochromatic histone modifications during replication leads to transient de-repression of repetitive sequences, and a closed chromatin state must be re-established [55], [56]. In budding yeast, heterochromatic silencing can be partially established during S phase, but mitosis is required to fully establish silencing [57], [58]. Such requirements may also apply to Drosophila, because elongation of the cell cycle by mutation [59] or by low temperatures [60] enhances silencing. Conversely, our observation that acceleration of the cell cycle de-represses silencing suggests that re-establishment is a slow process.

Cell cycle length may be important for heterochromatic function if silencing requires that heterochromatic closed chromatin states be duplicated every cell cycle. As chromatin duplicates in S phase, and associations between homologs are disrupted in mitosis, heterochromatin at the *bw^+^* locus must be re-established in this interval. Euchromatin and heterochromatin replicate in early and late S phase, respectively, and this temporal separation is important for maintaining the hypo-acetylation of heterochromatin [61]. The *bw^+^* locus may be silenced if pairing with *bw^D^* forces it to replicate late and become hypo-acetylated. Alternatively, pairing with *bw^D^* may be necessary to add repressive histone modifications after DNA replication. Accelerated cell cycles may drive early replication of *bw^+^* or mitosis before heterochromatic marks are duplicated, leading to the loss of a closed chromatin state. Importantly, our results imply that re-establishment of a closed chromatin state must occur every cell cycle, and if re-establishment fails it cannot be restored.

Regardless of how accelerated cell cycles lead to de-repression, the appearance of de-repressed clones in an otherwise silenced population of cells indicates that once an open chromatin state is established, it is stably propagated through multiple cell cycles. Perhaps open chromatin states are inherently heritable, but simply never occur early in development in *bw^D^/bw^+^* animals. Indeed, developmental differences in silencing have been previously observed, where dividing cells show severe silencing that “relaxes” upon differentiation [43]. Alternatively, cells may normally switch between open and closed chromatin states throughout development, but rapid cell cycles might prevent establishment of a closed state from an open state. If acceleration of the cell cycle causes early replication and hyper-acetylation of the *bw^+^* locus, this could hinder heterochromatin formation. For example, methylation of histone H3 at lysine-9 is required for heterochromatic silencing, but is blocked by acetylation at this residue [62]. This antagonistic relationship between modifications at this residue may also imply that a third, unmarked chromatin state may affect the stability of silencing. In any case, as the loss of euchromatic modifications can take multiple cell divisions [63], open chromatin states may only slowly switch to a closed state.

Most models for epigenetic systems assume the silenced state is somatically heritable, and propose that heritability is conferred by self-associating properties of silencing proteins. However, silencing also requires continual re-establishment by nascent transcription of repetitive sequences that direct RNAi-dependent histone modifications after every round of chromatin duplication [56]. Our work makes it clear that active states can also be somatically heritable, and suggests that somatically heritable patterns need not imply special features of chromatin-associated proteins. Stable de-repression has also been observed with Polycomb-dependent regulatory elements [64], suggesting that heritability is a common property of chromatin-based silencing systems. Thus, inheritance of either open or of closed chromatin states may generate clonal patterns of gene expression during development.

## Materials and Methods

All crosses were grown at 25°C or 18°C on standard cornmeal medium. Stocks, mutations, and balancer chromosomes not described here are detailed in Flybase (www.flybase.org).

### GAL4 driver lines

The lines referred to as ‘*GMRGAL*’ and ‘*A5CGAL*’ are previously described *white*-deficient versions of drivers for late eye-specific and constitutive expression of GAL4, respectively [65]. For constitutive eye-specific expression, we used the *P*[*eyGAL, w^+^*]3-8 line, referred to as *eyGAL* [66]. For experiments with *In(1)w^m4^*, the *eyGAL* driver was destabilized using *TMS, P*[*Delta2–3*] to generate a *white*-deficient insertion that retained eye-specific expression of GAL4.

### Mis-expression insertion screens

We used *st* or *v^36f^* to eliminate all ommochrome pigments from the eye. In these backgrounds, bw^+^ cells appear red, while cells with bw^+^ silencing appear white. A preliminary screen was performed using a *w^+^*-marked P[*EP*]2339 (inserted at 59E) as a donor for mutagenesis. We crossed *P*[*EP*]2339*/CyO; st* virgins to *Dr/TMS, P*[*Delta2–3*] males, and then crossed individual Cy^+^ Sb male progeny to *GMRGAL bw^D^/CyO; st* females. Cy^+^ Sb^+^ progeny with enhanced or de-repressed bw^+^ silencing were recovered and mated to a *w^1118^* stock for extraction of new *P*[*EP*] insertions.

A second screen used the *y^+^*-marked *P*[*LA*] construct for mutagenesis. We first transposed *P*[*LA*]4 [17] onto *FM7i* using *P* transposase, and then used the resultant chromosome *FM7i*, *P*[*LA*]4.2 as a donor chromosome. We crossed y/*FM7i*, *P*[*LA*]4.2; *st* females to *Dr/TMS, P*[*Delta2–3*] males, and then crossed individual progeny B Sb males to *y; GMR bw^D^/CyO; st* females. Cy^+^ Sb^+^ y^+^ males with enhanced or de-repressed *bw^+^* silencing were recovered and mated to a *y* stock for extraction of new *P*[*LA*] insertions.

Candidate gene insertion lines were obtained from the Bloomington Drosophila Stock Center (Bloomington IN) and the Exelexis Stock Center (Boston MA) and tested for effects on *bw^D^* silencing in *v^36f^; GMRGAL bw^D^/+* males. To assess if effects were dependent on expression of a gene adjacent to the *P* element, each insertion from screens and candidate tests were re-assessed with GAL4 (*GMRGAL bw^D^/insertion*) and without GAL4 (*bw^D^/insertion*). Progeny from crosses were scored and photographed 3–4 days after eclosion as previously described [67].

### Identification of target sites

New insertion sites were mapped using inverse PCR according to published protocols [68]. Genomic DNA from balanced lines was purified and digested using *MspI* or *RsaI* restriction enzymes, ligated, and used for PCR amplification using the following primers: P[*LA*] 5′ ends, *LA(f).1*/*LA(r).1*; P[*LA*] 3′ ends, *Pry4*/*Pry1*, or *Sp6*/*Pry4*; P[*EP*] 5′ ends, *Pwht1*/*Plac1*; P[*EP*] 3′ ends, *Pry4*/*Pry1*. Products from all 3′ ends were sequenced using the nested primer *Spep1*, for *P*[*LA*] 5′ends using *LA(f)seq1*, and for *P*[*EP*] 5′ ends using *Plac1*. The gene responsible for effects on heterochromatic gene-silencing was inferred to be the nearest gene down-stream of the inducible promoter.

### Constructs used to characterize modifiers

We tested whether *P*[*LA*] modifiers of *bw^D^* silencing altered *w^m4^* silencing by crossing *In(1)w^m4h^; GMRGAL* females to each insertion line and scoring silencing in male progeny. Insertion-bearing progeny were divided into 5 ranks based on the extent of *w^m4^* silencing and compared to silencing in siblings carrying a dominant marker (CyO or *Sco* for chromosome *2* inserts, and *Sb* for chromosome *3* inserts). At least 40 flies were scored for each genotype, and assessed for statistical significance using Mann-Whitney U tests. To test if insertions affected RNAi-mediated gene-silencing, we crossed each insertion line to *GMRGAL; P*[*GMR-wIR*] [69] and scored *w^+^* expression. To determine if modifying effects of insertions required expression in dividing cells, we used the cyclin inhibitor p21 to block cell cycle progression in the *GMR* expression domain of the eye [50]. We crossed each insertion line to *v^36f^; GMRGAL bw^D^*; *P*[*GMR-p21,w^+^*] and scored silencing in male progeny.

### Eye disc cytology

Imaginal eye-antennal discs were dissected from late 3^rd^ instar larvae in PBS. For detection of apoptosis, discs were incubated in 5 µg/mL acridine orange/PBS for 5 minutes, and then imaged using FITC excitation and emission filters. For detection of mitotic cells, discs were fixed with 2% formaldehyde, and stained with antisera to the mitosis marker H3-S10-phosphorylation (Millipore).

## Supporting Information

Figure S1 Over-expression modifiers do not affect RNAi. *GMRGAL* induces over-expression from a mis-expression insertion and the *mini-w^+^* marker in the transgene, and *GMR-wIR* produces hairpin RNAs that knock-down the *w^+^* transcript through RNAi. Over-expression of *Orc6* does not alter heterochromatic silencing and was used as a control, where RNAi of *w^+^* is efficient. Knock-down of *mini-w^+^* with over-expression of *Rm62* appears more efficient, while knock-down with over-expression of *P[EP]Su25* is decreased. Other tested modifiers had no effect on RNAi knock-down.
(10.01 MB EPS)

Table S1 Genomic positions of modifier insertions.
(0.02 MB XLS)

Table S2 Candidate genes with no effect on silencing.
(0.02 MB XLS)
